# Editorial: Redox metabolism: a double edge sword sustaining the adaptive resistance to therapy in cancer

**DOI:** 10.3389/fonc.2023.1260233

**Published:** 2023-08-15

**Authors:** Barbara Marengo, Josè Pedro Friedmann Angeli, Cinzia Domenicotti

**Affiliations:** ^1^ Department of Experimental Medicine, General Pathology Section, University of Genoa, Genoa, Italy; ^2^ Rudolf Virchow Center for Integrative and Translational Bioimaging, Chair of Translational Cell Biology, University of Würzburg, Würzburg, Germany

**Keywords:** metabolic reprogramming, redox signaling, antioxidants, chemoresistance, radioresistance, ferroptosis

Metabolic reprogramming is a pivotal hallmark of cancer that contributes to therapy resistance (1). Cancer cells possess the remarkable ability to modify their metabolism, enabling them to regulate Reactive Oxygen Species (ROS) levels. Maintaining ROS at moderate levels facilitates cell survival and proliferation. Understanding the intricacies of cancer redox metabolism is crucial for identifying specific targets that can improve therapy efficacy. This Research Topic features three original research articles, two review articles, and one perspective article, which showcase recent advancements in this field. Collectively, these articles provide compelling evidence supporting the modulation of redox metabolism as a potent strategy to counteract adaptive resistance in cancer therapy.

In their review article, Min et al. delve into the intricate role of cysteine, both bound to and free from proteins, in cancer biology. Cysteine, an amino acid, participates in several metabolic pathways, including the synthesis of reduced glutathione (GSH), a major endogenous non-enzymatic antioxidant. Additionally, cysteine plays a crucial role in generating sulfur-containing biomolecules, such as hydrogen sulfide (H_2_S), taurine, coenzyme A, and biotin (2). Furthermore, the oxidation of cysteine residues in numerous phosphatases, kinases, and transcription factors can modulate their activities, impacting cancer cell survival and therapy resistance. Given the pro-oxidant nature of most chemotherapeutic drugs, there is a growing interest in developing covalent inhibitors that specifically target cysteine residues near the ATP-binding pocket of redox signaling proteins. Moreover, GSH has been widely acknowledged for its crucial role in cancer progression and therapy resistance (3–5). Pompella et al.‘s perspective article highlights the interaction of glycyl-cysteine, a dipeptide originating from GSH metabolism, with cisplatin. This interaction impedes cisplatin’s access to cancer cells, reducing its cytotoxic efficacy. GGT1 expression emerges as a significant biomarker for cisplatin resistance. Notably, cisplatin-induced effects are mediated by ROS overproduction and lipid peroxidation (6), underscoring the need to investigate GGT1’s potential to prevent ferroptosis, a form of programmed cell death induced by GSH depletion and resulting in peroxidation of membrane phospholipids (7, 8). The involvement of high intracellular GSH levels in therapy resistance is further confirmed by Garbarino et al.‘s original research article, demonstrating that BRAF-mutated metastatic melanoma cells develop resistance to PLX4032 (Vemurafenib), a BRAF inhibitor approved for melanoma treatment (9). This resistance is attributed to a metabolic rewiring of oxidative phosphorylation and the maintenance of pyruvate dehydrogenase activity. Leveraging an *in vitro* model of drug resistance derived from patient-isolated melanoma cells, the authors propose that inhibitors targeting GSH biosynthesis and/or pyruvate dehydrogenase activity, in combination with PLX4032, could overcome therapy resistance.

In response to mounting evidence implicating redox metabolism as a critical regulator of tumor progression and therapy response, Ji et al. have developed a novel constraint-based computational method, COSM^ro^, to integrate redox signaling and metabolic networks. Applying this innovative approach to a head and neck cancer model of radiation resistance, the authors uncover a relationship between intracellular redox state and cholesterol metabolism. Intriguingly, their network analysis highlights pathways potentially contributing to radioresistance, including the activation of the phosphatidyl-inositol-3-kinase/protein kinase B (AKT) pathway and the rerouting of glycolysis into the pentose phosphate pathway (PPP) for generating essential molecules like nicotinamide adenine dinucleotide phosphate (NADPH) and nucleotide building blocks crucial for repairing radiation-induced DNA damage. Complementing this research, Goetting et al. found that the aberrant activation of AKT exerts an influence on cell metabolism, antioxidant defense, and radiosensitivity. The study reveals that cancer cells expressing the AKT variant (AKT-E17K) exhibit radioresistance correlated with enhanced GSH levels through novel GSH biosynthesis promotion and increased GSH regeneration *via* NADPH formation. Numerous studies have established the involvement of nuclear factor (erythroid-derived 2)-like 2 (Nrf2) in the antioxidant response and metabolic switch, which drives the adaptive response of cancer cells to chemo and radiotherapy (10–12). Inhibitors of Nrf2 have demonstrated their potential in sensitizing various types of cancer cells to these therapies. Fascinatingly, Oronsky et al. report on the triple action of RRx-001, a nonpolar small molecule that readily penetrates cell membranes and the blood-brain barrier. RRx-001 acts as an NLRP3 inflammasome inhibitor, nitric oxide superagonist, and Nrf2 inducer. It can switch between pro-oxidant/pro-inflammatory and antioxidant/anti-inflammatory activities, depending on the redox state and tissue oxygenation.

Collectively, the articles included in this Research Topic strengthen the pivotal role of redox metabolism in determining cancer cell resistance to therapy and although further studies are needed, we hope that information herein collected can be helpful to spark more effective and personalized anticancer strategies to improve patient’s outcome and survival. We would like to thank all the authors who have participated in the realization of this Research Topic and shared their knowledge and the latest results of their studies. Finally, we would like to thank the reviewers for objectively evaluating the submitted papers and the editorial staff for their kind support.

## Author contributions

BM: Writing – original draft, Writing – review & editing, Writing – original draft, Writing – review & editing. JA: Writing – original draft, Writing – review & editing, Writing – original draft, Writing – review & editing. CD: Writing – original draft, Writing – review & editing, Writing – original draft, Writing – review & editing.
